# The role of macrophage and adipocyte mitochondrial dysfunction in the pathogenesis of obesity

**DOI:** 10.3389/fimmu.2024.1481312

**Published:** 2024-11-08

**Authors:** Min Wang, Min Min, Haojie Duan, Jia Mai, Xiaojuan Liu

**Affiliations:** ^1^ Department of Laboratory Medicine, West China Second University Hospital, Sichuan University, Chengdu, Sichuan, China; ^2^ Key Laboratory of Birth Defects and Related Diseases of Women and Children (Sichuan University), Ministry of Education, Chengdu, Sichuan, China; ^3^ Outpatient Department, The Air Force Hospital of Western Theater, PLA, Chengdu, Sichuan, China

**Keywords:** macrophages, mitochondrial disorder, adipose tissue, obesity, pathogenesis

## Abstract

Obesity has emerged as a prominent global public health concern, leading to the development of numerous metabolic disorders such as cardiovascular diseases, type−2 diabetes mellitus (T2DM), sleep apnea and several system diseases. It is widely recognized that obesity is characterized by a state of inflammation, with immune cells-particularly macrophages-playing a significant role in its pathogenesis through the production of inflammatory cytokines and activation of corresponding pathways. In addition to their immune functions, macrophages have also been implicated in lipogenesis. Additionally, the mitochondrial disorders existed in macrophages commonly, leading to decreased heat production. Meantime, adipocytes have mitochondrial dysfunction and damage which affect thermogenesis and insulin resistance. Therefore, enhancing our comprehension of the role of macrophages and mitochondrial dysfunction in both macrophages and adipose tissue will facilitate the identification of potential therapeutic targets for addressing this condition.

## 1 Introduction

Obesity is a chronic metabolic disease whose main characteristic is harmful overweight and the accumulation of adipose tissue (AT). The WHO definition of obesity is a BMI≥30 kg/m^2^ (1). For several decades, the prevalence of obesity has increased significantly. According to a recent study published in *Lancet*, there were 159 million obese children and adolescents, and 879 million obese adults globally in 2022. The global obesity rate for children and adolescents in 2022 was approximately four times greater than that in 1990. Among adults, obesity rates have nearly tripled in males and more than doubled in women (2). Frequently, obesity is related to a variety of metabolic diseases, such as insulin resistance-induced type 2 diabetes and cardiovascular disease (CVD) (3, 4). Compared with healthy individuals, obese individuals have a greater hazard ratio for cancers of the breast, kidney, pancreas, and esophagus (5, 6). Moreover, childhood obesity is closely correlated with metabolic diseases in adulthood, so prevention of childhood obesity is essential (7).

The basic cause of obesity is an imbalance between caloric intake and consumption; this situation is usually caused by an excess high-fat diet and a lack of physical activity (8). Therefore, common individual management strategies for the prevention of obesity include reducing the consumption of high-carbohydrate or high-fat diets and increasing the frequency of workouts. By doing so, a weight loss of 5% to 7% can be produced on average, which is not desirable or sufficient (9). Under conditions of obesity, several alterations occur, including metabolic pathways activation related with energy metabolism, proinflammatory cytokine upregulation, immune cell population changes, and mitochondrial dysfunction (10–13). Currently, various anti-obesity medications target these alterations to control energy balance and inhibit inflammation, thus achieving the goal of weight loss and reducing the corresponding disease risks (14).

As the most abundant immune cell type in adipose tissue, macrophages play crucial roles in maintaining adipose homeostasis and regulating the immune system (15). Not only the number but also the tissue localization and phenotype of macrophages are largely altered during the process of obesity (12). Macrophages in adipose tissue are the principal source of inflammatory mediators which contribute largely to the production of chronic low-grade inflammation in obese individuals (16, 17). In addition to their immune functions, macrophages have also been implicated in lipogenesis. Additionally, mitochondrial disorders commonly exist in macrophages, leading to decreased heat production in adipose tissue. Moreover, adipocytes exhibit mitochondrial dysfunction and damage, which affect thermogenesis and insulin resistance. Mitochondrial disorders can act as a bridge that connects macrophages and adipocytes, together contributing to the development of obesity. In this review, we summarize both macrophages and adipose tissue mitochondrial disorders in adipose tissue.

## 2 Overview of obesity-associated factors

Obesity is the result of energy input being greater than energy output, and then excess energy is converted into fat under the subcutaneous or internal organs. The factors that induce obesity can be divided into congenital factors, postnatal factors, and environmental factors. Congenital factors mainly refer to related gene expression. During the process of evolution, those who could store and utilize energy more effectively and tolerate hunger for longer may have a comparative reproduction advantage over those who do not have these properties according to survival and selection stress, thus resulting in overrepresentation of genetic variants that are beneficial for faster eating, excess energy absorption and storage in adipose tissue (18).

Many studies have investigated genes associated with obesity. For example, fat mass and obesity-associated genes (FTOs) were identified as obesity markers by genome-wide association studies, and FTO knockout mice exhibited weight loss (19). Additionally, with the help of whole-exome sequencing, a series of genes were identified as BMI-related genes. For example, G-protein coupled receptor (GPR75) was associated with a lower BMI, whereas calcitonin receptor (CALCR) was correlated with a higher BMI (20). Other sequencing analyses focusing on sex and age differences also revealed that several genes function in men and women or adults and children separately (21). Moreover, mutations in genes encoding adipose tissue hormones and hormone receptors play a significant role in the development of obesity (22–25). Findings related to these genes not only help explain the probable mechanism of obesity occurrence but also offer potential therapeutic targets for obesity.

Excessive food intake, unhealthy eating habits, insufficient physical activity, a lack of sleep, psychological and emotional changes, region and socioeconomic status are all influencing factors of overweight (8, 26–28). In addition to these factors, immune and metabolic status are critical propellants in promoting obesity because adipose tissue is not only an energy bank but also an endocrine organ (29). Innate immune cells, including macrophages, lymphocytes, dendritic cells, neutrophils, eosinophils and natural killer cells, which reside in adipose tissue can secrete numerous cytokines that affect metabolic pathways and facilitate inflammation (16, 30–33).

## 3 Adipose tissue macrophages

Obesity is actually a state of chronic inflammation, and the infiltration and recruitment of adipose tissue macrophages (ATMs) greatly contribute to the process of obesity. Macrophages are a group of cells that are differentiated from monocytes and have the ability to phagocytose cell debris and pathogens. Generally, there are two principal macrophage populations. The classically activated status is type 1 macrophages (M1 macrophages), which usually express CD11c, TNF, IL-6, IL-1β, and Nos2. They are commonly activated by IFN-γ, LPS and GM-CSF, and secrete proinflammatory cytokines (34–36). The alternatively activated type is type 2 macrophages (M2 macrophages), which express arginase 1(Arg1), CD206, and CD301 (37). M2 macrophages are usually stimulated by M-CSF, IL-13 and TGF-β, and they secrete anti-inflammatory cytokines, including IL-1RA and IL-10 (38). M2 macrophages can be divided into four subtypes according to different stimuli and cell markers. M2a cells, also called alternative activated cells, can be activated by IL-4 and IL-13 and they highly express CD206, CD209 and FIZZ1. Type II alternatively activated cells, commonly called M2b cells, are usually activated by IL-1β or LPS and they highly express CD80 and CD14. M2c macrophages are also referring to acquired deactivated cells; they can be stimulated by TGF-β and IL-10 and highly express CD163 and CD206. M2d macrophages, also called tumor-associated macrophages (TAMs), are induced by TLR agonists, and they may participate in the proliferation and invasion of tumor cells (38–40). In fact, the number, phenotype, and tissue localization of macrophages can be significantly altered as BMI increases.

In addition to the two classic cell subtypes, other macrophage subtypes are also associated with adipose tissue. In a previous study, CD11c^-^CD206^-^ ATMs were recognized as type 3 macrophages, which localize to crown-like structures (CLSs) and express pro-inflammatory cytokines (41, 42). CD11c^+^CD206^+^ ATMs are in the middle state of M1/M2 ATMs, which have relatively high levels of lipid-rich vacuolar and mitochondrial RNAs, as well as transcripts encoding APOE, FABP4, and fatty acid metabolism enzymes (43). 11β-hydroxysteroid dehydrogenase type 1(11β-HSD1) is a reductase which catalyzes inactive glucocorticoids into active form (44). One study revealed that the 11β-hydroxysteroid dehydrogenase type 1–glucocorticoid receptor (11β-HSD1-GR) regulatory axis plays an important role in the process of switching to the M1/M2 phenotype and prevention of this process may be a potential therapeutic target for obesity (45).

Lipid-associated macrophages (LAMs) are a subtype of CD11c^+^CD206^+^ ATMs. LAMs express a series of transcriptional genes associated with lipid embolism and phagocytosis, including Trem2, Lipa, and Ctsb. In particular, Trem2, which is highly expressed in LAM, plays a crucial role in ATM remodeling, prevention of adipocyte hypertrophy and maintenance of metabolic homeostasis (46). Another subtype of lipid-laden macrophages named CD9^+^ macrophages, which localize to CLSs, express several genes related to lysosomal pathways and proinflammatory mediators (47).

**Figure 1:**
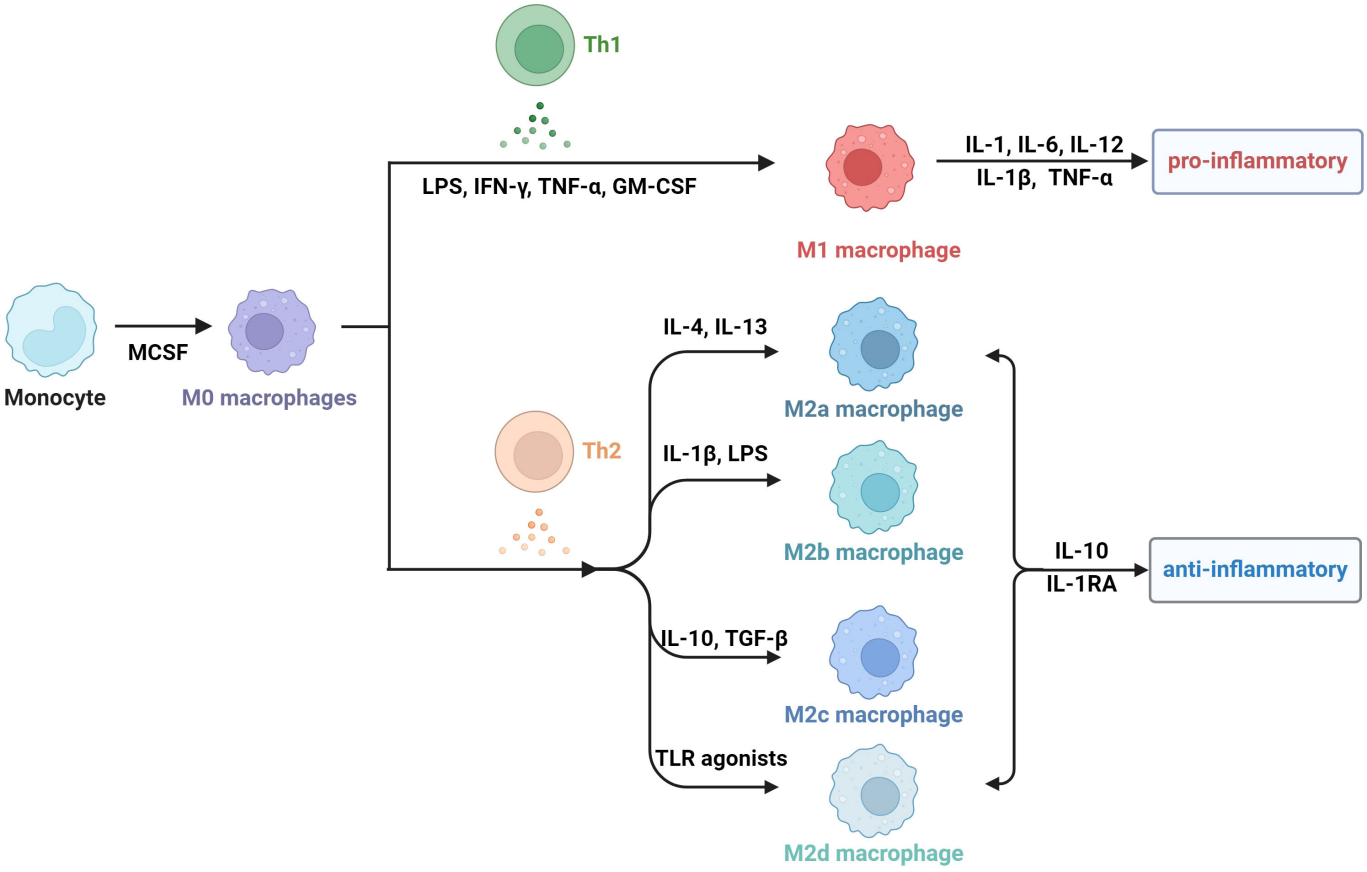
The subtype classification of macrophages.

Additionally, a metabolically activated macrophage (MMe) phenotype, which overexpresses ABCA1, CD36 and PLIN2 but does not express M1 cell surface markers including CD38, CD319 and CD274, and M2 cell surface markers including CD163 and CD206, is produced under stimulation with saturated free fatty acids (FFAs) (48). (**Table 1**) Excessive lipids can polarize macrophages toward an inflammatory state and induce lysosome biogenesis in macrophages, which helps lipid clearance. The inhibition of ATM lysosomal activity inhibits lipid metabolism, increases lipid accumulation in ATMs, and decreases overall AT lipolysis (49). Dead adipocytes are a plentiful source of FFAs, which are essential for metabolic activation, and MMe can clear these dead cells through lysosomal exocytosis with the help of TLR2, NOX2, and MYD88 (50) (**Figure 1**).

**Table 1:** Macrophages subtypes related with obesity.

Type of macrophages	Cell marker	Function	Reference
Lipid-associated macrophages (LAM)	Trem2, Lipa, Ctsb	ATM remodeling	(46)
CD9^+^	CD9	Related with lysosomal pathways and proinflammatory mediators	(47)
metabolically activated macrophage (MMe)	ABCA1, CD36, PLIN2	clear dead cells through lysosomal exocytosis	(48)

## 4 Regulatory factors governing macrophage polarization

Macrophage polarization is crucial for inflammation and obesity. There are various regulatory factors governing macrophage polarization.

As one of the most important inducers of M1 polarization, LPS stimulation increases the expression of M1 macrophage markers, such as toll-like receptor 4 (TLR4), CD36, and CD68 (51). It can interact with TLR4, together inducing production of TRIF, TRAM and Myd88, and the former two can recruit TANK through tumor necrosis factor receptor-associated factor 3 (TRAF3), then activate and combine with TANK-binding kinase 1 (TBK1), thus activating interferon-regulatory factor 3(IRF3), while Myd88 activates NF-κB pathway (52). Additionally, IFN-γ secreted from Th1 cells, NK cells and CD8^+^ T cells can activate Janus kinase 1(JAK1) and Janus kinase 2 (JAK2), consequently leading to the activation of signal transducer and activator of transcription 1(STAT1), which together promote M1 polarization (53). Similarly, GM-CSF contributes to M1 polarization through JAK2-STAT5 signaling pathway (54). Except these molecules, hormones also participate in the process. For example, Leptin is a protein encoded by ob genes which is generally thought to be a proinflammatory cytokine (55). Leptin/obR significantly activated M1 macrophages via JNK/STAT3/AKT signaling and CXCL2 production (56). Usually, obesity leads to an increase in proinflammatory T cells, including CD4^+^ Th1 and CD8^+^ effector T cells, whereas anti-inflammatory Th2 cells decrease in adipose tissue, which induces macrophage polarization toward the M1 phenotype (57). And leptin can influence the polarization of CD4^+^ T cells toward the Th1 phenotype by enhancing Th1 responses and suppressing Th2 immunological responses (58). Importantly, diet-induced obesity increases the number of NK cells and causes them to release IFN-γ and TNF-α in visceral adipose tissue, amplifying polarization and infiltration of M1 macrophage (59).

Correspondingly, there are a series of factors involved in M2 polarization. IL-10 promotes gene expression associated with an M2-like phenotype, and this process was determined to be STAT3 dependent through JAK1 activation (60, 61). IL-3 can activate JAK2 followed by STAT5 recruitment, leading to M2 polarization (62, 63). The binding of IL-4 and IL-13 to the corresponding receptor activates STAT6 through JAK1 and JAK3, finally activates IRF4 causing macrophages to undergo M2 polarization (64). Besides, adiponectin, a 247 amino acid protein, mainly plays a role in metabolism control by promoting glycolysis and fatty acid oxidation while inhibiting gluconeogenesis (65). It can regulate M2 macrophage polarization through activating the jumonji domain-containing - 3 (JMJD3)-IRF4 axis (66). Furthermore, adiponectin can affect the secretory function of macrophages, which is demonstrated by reduced secretion of proinflammatory cytokines such as IL-6 and TNF from macrophages and increased production of anti-inflammatory cytokines, including IL-10 and IL-1RA (67).

**Figure 2:**
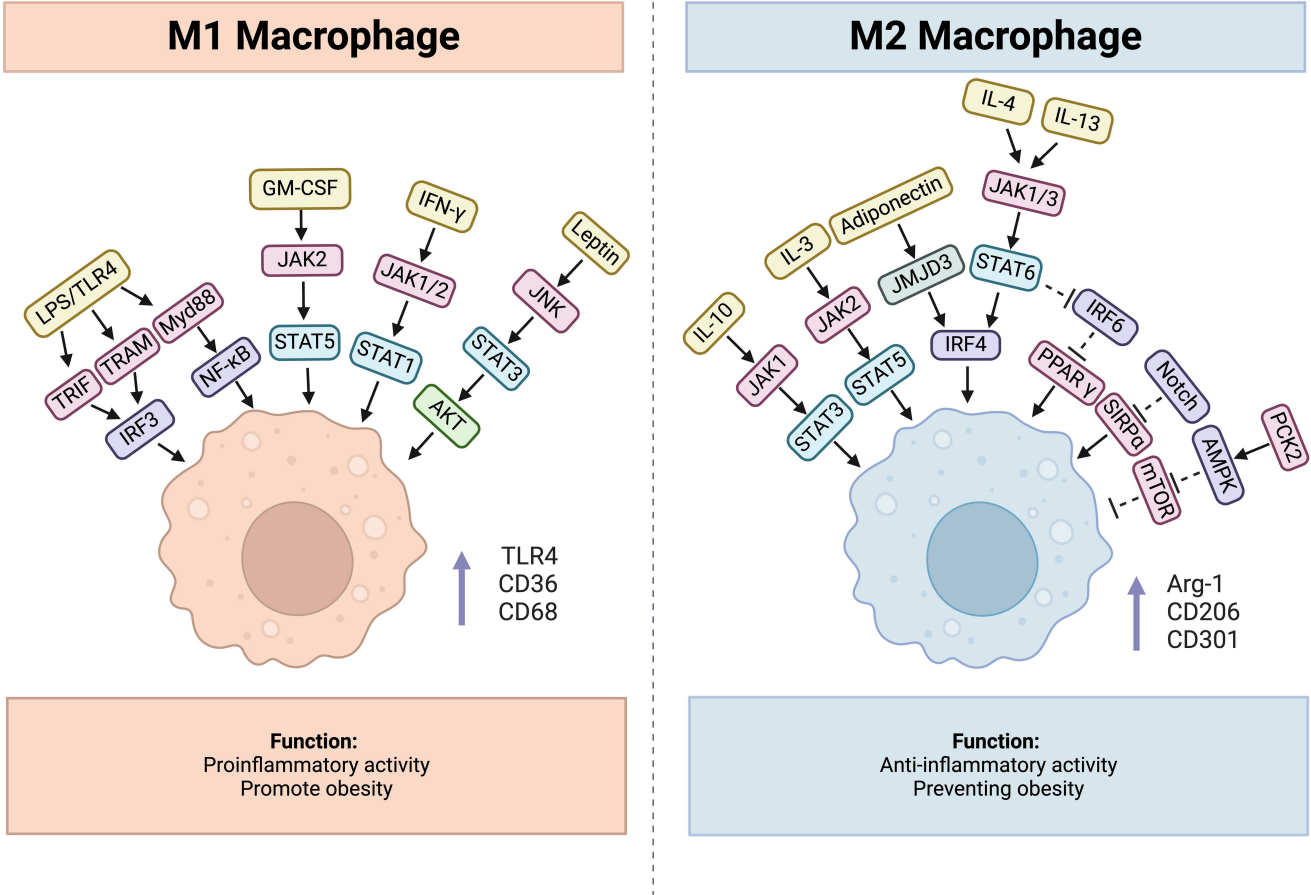
Factors influencing process of macrophage polarization, cytokines, hormones, and related signaling pathways are included.

Besides, IRF6 reduces M2 polarization by binding to the peroxisome proliferator-activated receptor γ (PPARγ) promoter and limiting its expression, while the JAK1/3-STAT6 pathway can inhibit IRF6 expression (68). Signal regulatory protein α (SIRPα) has been demonstrated to promote M2 polarization, whereas the Notch pathway can activate M1 polarization through the suppression of SIRPα expression (69). There also exists a phosphoenolpyruvate carboxykinase 2(PCK2)- AMP-activated protein kinase (AMPK)- mammalian target of rapamycin(mTOR) pathway regulating M2 polarization (70). AMPKα1 and AMPKβ1 are both important for M2 polarization, and AMPKβ1 deletion in macrophages decreases the mitochondrial content and rate of FAO (71, 72). (**Figure 2**)

Overall, inhibiting factors polarizing macrophages toward M1 and facilitating factors polarizing macrophages toward M2 can be therapy targets for obesity.

## 5 Regulatory factors governing macrophage recruitment

Macrophage recruitment plays a crucial role in promoting obesity and related inflammation. Compared with lean mice, obese mice exhibit increased infiltration of macrophages, accounting for 40% of all AT cells in obese mice (73). 90% of the macrophages that infiltrate the adipose tissue of obese humans and animals create characteristic CLSs surrounding dead adipocytes (74). Saturated fatty acids released by adipocytes and proinflammatory cytokines such as MCP-1 and TNF-α secreted from macrophages can lead to macrophage recruitment (75). Together, these factors augment inflammation in obesity and increase insulin resistance.

Monocyte chemoattractant protein-1 (MCP-1) is a proinflammatory chemokine that is produced largely by macrophages and endothelial cells (76). C-C motif chemokine receptor-2 (CCR2) is the receptor of MCP-1 (also called CCL2). The main function of MCP-1/CCR2 is regulating monocyte and macrophage recruitment. MCP-1 levels in plasma increased in both genetically obese diabetic (db/db) and WT mice with obesity caused by a high-fat diet, leading to macrophage infiltration. Conversely, MCP-1 gene knockout reduces macrophage accumulation in adipose tissue (77). Therefore, inhibiting the expression of MCP-1, CCR2, or other factors that can regulate the levels of MCP-1 may provide potential therapeutic methods for obesity.

Other chemokines, such as C-X-C motif receptor 3 (CXCR3) and C-X-C motif chemokine receptor 7 (CXCR7), are involved in the recruitment of macrophages to adipose tissue. In Th1 cells, CXCR3 combines with its ligands CXCL9 and CXCL10 to produce memory and effector T cells (78). Mice fed a high-fat diet presented increased numbers of CXCR3-expressing CD8^+^ T cells and IFN-γ-expressing CD4^+^ T cells. These cells attract and polarize macrophages toward an M1 response, leading to chronic low-grade inflammation during obesity (79). CXCR3^-/-^ mice presented a decreased VAT macrophage response, and CXCR3^-/-^ macrophages presented a defective response to LPS, resulting in a reduction in IL-12 and TNF-α synthesis *in vitro (* 80). Therefore, targeting the CXCR3 receptor may be a potential treatment for obesity. Additionally, CXCR7 is expressed in adipose tissue, and its ligands CXCL11 and CXCL12 mediate macrophage chemotaxis and phagocytosis and contribute to inflammation during obesity (81, 82). In obesity, the expression of CXCR7, CXCL11 and CXCL12 is increased, and CXCR7 neutralizing therapy with an anti-CXCR7 antibody can not only reduce macrophage infiltration and inflammation in obesity but also improve insulin resistance (83).

## 6 ATMs influence lipid metabolism and the energy state

The main function of adipose tissue is energy storage, and adipose tissue is divided into white adipose tissue (WAT) and brown adipose tissue (BAT). WAT, including subcutaneous adipose tissue (SAT) and visceral adipose tissue (VAT), plays an important role in storing excessive lipids as triglycerides and releasing free fatty acids (FFAs) in the state of hunger. In contrast, BAT is rich in mitochondria, which can divert ATP into heat through uncoupling protein 1 (UCP1) and uncoupling electron transport to maintain temperature balance and fight against obesity (84). In fact, beige adipose tissue, similar to BAT, has heat production ability (85). Since the energy consumption of BAT and beige adipose tissue is greater than that of WAT, increasing the percentage or promoting the browning process of BAT and beige adipose tissue may be a potential therapy to combat obesity. For example, succinate is an intermediate metabolite of the tricarboxylic acid cycle that plays an indispensable role in energy production via the mitochondrial pathway. The increasing circulating level of succinate in BAT and beige fat increases whole-body energy expenditure, exacerbates obesity and inhibits systemic tissue inflammation. Mechanistically, succinate affects BAT mitochondrial or mitochondria-related proteins, which can help promote body weight loss (86). However, adipocyte disorders occur in obesity, and adipocytes lose their heat production ability, resulting in the accumulation of dead adipocytes. The clearance of these dead cells requires macrophages.

In fact, macrophages function as important members of intrinsic immunity and can influence metabolic pathways and metabolites in adipose tissue. One of the most significant features of macrophages in obesity is the upregulation of lysosome-related pathways. A previous study revealed that the surface marker LAMP-1, which indicates lysosome exocytosis, is increased in CLS macrophages (50). In addition, macrophages can absorb fat from dead adipocytes through an acidified interface between adipocytes and macrophages. If lysosomal function in ATMs is hindered, lipid metabolism is affected, and lipid accumulation increases (49). Legumain (Lgmn), a typical lysosomal cysteine protease, is highly expressed in macrophages in response to overfeeding. Lgmn generated from macrophages can inhibit PKA activation and reduce the expression of lipolysis-related proteins through combination with integrin α5β1 in adipocytes, whereas Lgmn-knockout macrophages can regulate lipid metabolism and alleviate insulin resistance (87). However, as evidenced by a previous study, when injected with exogenous recombinant Lgmn, the body weight and food intake of the mice were significantly reduced (88). Overall, if macrophage function is impaired, dead fat cells cannot be recycled, and lipolysis decreases.

## 7 Alterations in mitochondrial biogenesis in ATMs

To achieve lipolysis, macrophages require significant energy expenditure to complete the process. However, in obesity, alterations in mitochondrial biogenesis occurring in ATMs lead to macrophage dysfunction. Inflammatory macrophages exhibit increased glycolytic metabolism and decreased mitochondrial oxidative phosphorylation (89). Many factors and mechanisms are involved in the acquisition of mitochondrial metabolic adaptations by macrophages. Fatty acid oxidation (FAO) mainly occurs in the mitochondrial matrix, thus producing ATP. FAO is closely related to M2 polarization. Macrophage programs for mitochondrial biogenesis and fatty acid oxidation are induced in response to IL-4, PPARγ-coactivator-1β (PGC-1β), and signal transducer and activator of transcription 6 (STAT6) (90). During inflammasome-mediated inflammation, histone deacetylase 3 (HDAC3) translocates to mitochondria, leading to deacetylation and a decrease in the activity of HADHA (mitochondrial trifunctional enzyme subunit α) - a key enzyme in mitochondrial fatty acid oxidation, which helps macrophages acquire FAO and mitochondrial morphology adaptations and ultimately promotes IL-1β production (91). In addition, as a member of the PGC-1 family, PGC-1β plays crucial roles in adaptive thermogenesis and mitochondrial fatty acid oxidation (92). As evidenced by a previous study, palmitic acid-induced TNF-α, MCP-1, and IL-1b mRNA and protein expression are reduced by PGC-1β, which can inhibit TAB1/TAK1 complex formation and TAK1 activation, thus decreasing macrophage-induced inflammation (93). Synoviolin (Syvn1), an E3 ubiquitin ligase, is an important target of inflammatory cytokines such as TNF-α, IL-1 and IL-17 (94). Syvn1 can interact with and ubiquitinate PGC-1β, and Syvn1 deficiency results in decreased weight and lipid accumulation. Mechanistically, the expression of PGC-1β target genes is upregulated along with increasement in respiration, basal energy expenditure and the quantity of mitochondria when syvn1 knocked out (95). Additionally, when macrophages are stimulated innately, mitochondrial ROS are produced. These ROS trigger the activation of Fgr kinase, which controls complex II activity and leads to macrophage polarization. The absence of Fgr leads to increased FAO and decreased lipid droplet accumulation after exposure to pathogen-associated molecular patterns (96).

In addition to immune functions such as phagocytosis and cytokine secretion, macrophages are also involved in fat storage and utilization. Many studies have demonstrated that macrophage-deficient mice exhibit weight loss and lean conditions. For example, colony stimulating factor 1 receptor (CSF1R) deletion can eliminate macrophages, resulting in the loss of visceral adipose tissue in rats (97). Additionally, macrophages can regulate energy storage and usage by molecules. Adipose-tissue resident macrophages can secrete PDGF family growth factors to mediate lipid storage. In the absence of PDGFcc, mice exhibit lean conditions through the conversion of extra lipids to thermogenesis or ectopic accumulation (98). Slc6a2 is a norepinephrine (NE) transporter expressing on sympathetic neuron-associated macrophages (SAMs). When Slc6a2 knocked out in SAMs, weight loss in obese mice is significant and consistent, and the proportion of brown adipose tissue is increased due to the decreased clearance of NE, which promotes adaptive thermogenesis and lipid mobilization (99). These results suggest that macrophages play a role in energy storage.

## 8 Alterations in mitochondrial biogenesis in adipocytes

In addition to changes in mitochondrial biogenesis in macrophages, mitochondrial changes also occur in adipocytes. As the center of metabolism, the mitochondrion is indispensable for survival because it is the main location for aerobic respiration and ATP production. The development and maturation of adipocytes are influenced by mitochondrial function. Early mitochondrial metabolism, biogenesis and ROS production are essential for promoting adipocyte differentiation in an mTORC1-dependent manner (100). Excessive nutrient consumption has been linked to mitochondrial dysfunction (101). Compared with lean people, obese people have mitochondria with reduced oxidation of fatty acids, less defined internal membranes and lower energy generating capacity (102, 103). In addition, adipocytes from high-fat diet-fed mice undergo mitochondrial fragmentation, which reduces their oxidative capacity through a mechanism mediated by the small GTPase RalA, whereas when RalA is deleted, decreased energy expenditure and mitochondrial oxidative phosphorylation are rescued (104). In addition, a study revealed that the interaction substrate of the transcription factor Parkin was accumulating in adipose progenitor cells from obese mice, which suppressed the expression of peroxisome proliferator-activated receptor γ coactivator-1α (PGC-1α) - a crucial regulator of mitochondrial biogenesis (105).

## 9 Mitochondria as a bridge connecting ATMs and adipocytes

**Figure 3:**
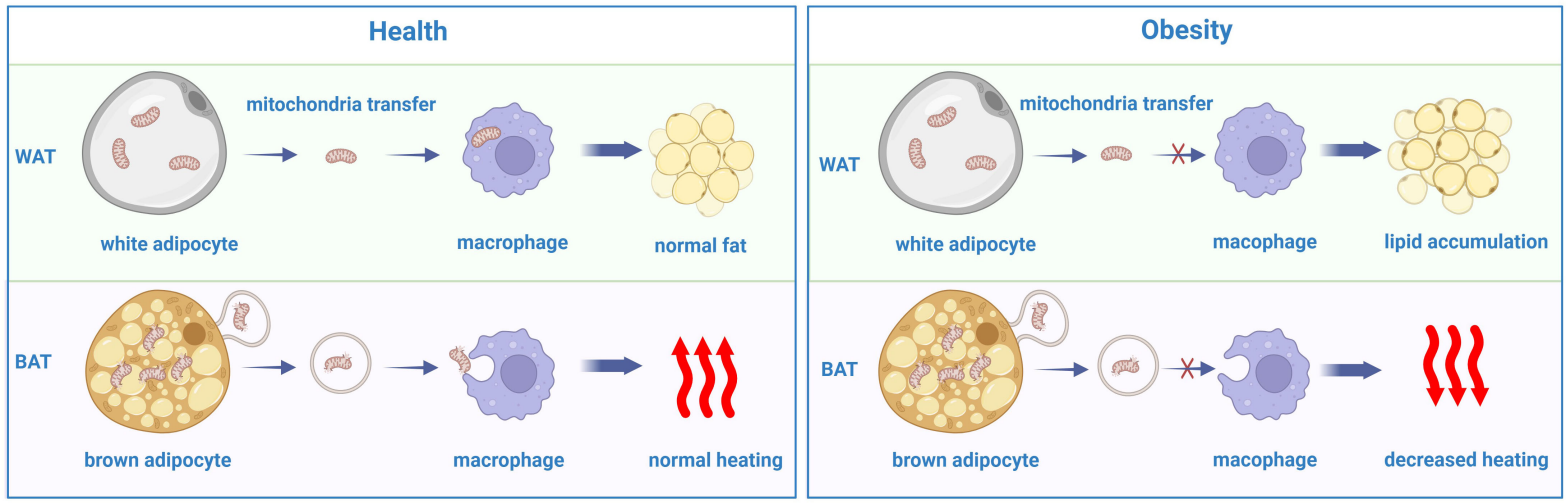
The disorder of mitochondrial transfer between adipocytes and macrophages in obesity comparing with the heath.

Previous studies have demonstrated that mitochondria can be transferred between cells to help metabolically challenged cells survive and intercellular mitochondrial transfer is related to several diseases, such as ischemic stroke, allograft rejection and the development of some cancer cells (106–109). Recently, a study revealed that intercellular mitochondrial transfer also occurs in WAT. Macrophages in WAT can acquire mitochondria from adipocytes *in vivo*, and when Ext1, a gene required for mitochondrial transfer, is genetically deleted, the fat storage increases, whereas energy expenditure decreases (110). A lard-based high-fat diet can inhibit the absorption of mitochondria by macrophages in white adipose tissue while diverting mitochondria released from adipocytes to other organs via the blood (111). In addition, brown adipocytes under thermogenic stress release extracellular vesicles (EVs) containing mitochondrial fragments that have been oxidatively damaged. When reabsorbed by parental brown adipocytes, mitochondria-derived EVs decreased the levels of UCP1 and PPARγ signaling. BAT-resident macrophages eliminate them, which is essential for maintaining BAT function. The aberrant buildup of extracellular mitochondrial vesicles in BAT results from the depletion of macrophages *in vivo*, which inhibits the body’s natural thermogenic response to cold exposure (112) (**Figure 3**).

The secretion of the macrophage cytokine Slit3 from ATMs increased the mRNA levels of UCP1, PGC1α, PRDM16, PPARγ, and Cycs, all of which are associated with thermogenesis. Slit3-overexpressing M2 macrophages can be transferred to WAT, which promotes beiging and thermogenesis (113). Additionally, proinflammatory cytokines secreted from macrophages can affect adipocyte thermogenesis. When cocultured with conditioned media generated from RAW macrophages, the UCP1 mRNA expression level was inhibited in C3H10T1/2 adipocytes. One study demonstrated that TNF-α inhibited transcription factors that bind to the cAMP response element and the UCP1 promoter, thus leading to UCP1 downregulation (114). Additionally, as the master proinflammatory cytokine, the expression of IL-1β is increased in inflammation and obesity. In adipocytes, isoproterenol-induced upregulation of UCP1 can be hindered by IL-1β, which is increased in obese WAT through the activation of extracellular signal-related kinase (ERK). Furthermore, the adipose tissues of mice treated with IL-1β substantially impaired the activation of UCP1 in response to cold (115). In addition to proinflammatory cytokines, anti-inflammatory cytokines are involved in the process of adipocyte thermogenesis. IL-10 usually has an anti-inflammatory effect; however, when IL-10 is ablated, mice exhibit anti-obesity traits, including increased energy expenditure and adipose thermogenesis. Mechanically, IL-10 changes the chromatin architecture and associated transcription factors of thermogenic genes (116). Another cytokine, IL-27 targets adipocytes directly through activating p38 MAPK-PGC-1α signaling and inducing UCP1 synthesis, which makes it a potential therapeutic target for obesity (117). Furthermore, as an anti-inflammatory cytokine, IL-4 can inhibit lipid accumulation and increase the expression of UCP1 in white adipose tissue (118). Altogether, mitochondria can be transferred between macrophages and adipocytes, and cytokines secreted from macrophages can also influence energy consumption in adipocytes.

## 10 Therapy targets

obesity is an imbalance of energy intake and consumption, actually there is mitochondrial dysfunction in adipocytes, so alleviating mitochondrial damage and increasing thermogenesis are also energy expenditure methods. In addition, obesity is a state of inflammation, and macrophages participate largely in the inflammatory process; thus, decreasing infiltration and hindering the polarization of macrophages is also a major direction of therapy.

### 10.1 Targeting macrophage recruitment and polarization

Macrophage polarization contributes significantly to the development of obesity. Ubiquitin-specific proteinase 14 (USP14) is highly expressed in ATMs of obese human patients and diet-induced fat mice and can aggravate macrophage recruitment and polarization. However, pharmacological inhibition of USP14 effectively reduces diet-induced hyperlipidemia and insulin resistance in mice, making it an important restraint on the proinflammatory M1 phenotype and therefore limiting obesity-related metabolic disorders (119). Additionally, neuregulin 4 belonging to the epidermal growth factor family is abundant in brown adipose tissue. It reduces inflammation by increasing M1 macrophage death and decreasing inflammatory factor release (120). Sirtuin 3 is a mitochondrial deacetylase that comprehensively participates in the regulation of mitochondrial biology. In ATMs from mice feeding a high-fat diet, sirtuin 3 levels were notably decreased. The proinflammatory macrophage polarization caused by palmitic acid was worsened by SIRT3 inhibition or knockdown. Mechanistically, the absence of SIRT3 caused hyperacetylation of succinate dehydrogenase, which in turn caused succinate accumulation. This buildup suppressed the transcription of kruppel-like factor 4 by increasing the level of histone methylation on its promoter, thereby inducing proinflammatory macrophages (121). These findings suggest the protective role of SIRT3. As a member of the adiponectin paralog family, C1q/tumor necrosis factor-related protein 6 (CTRP6) may influence macrophage glycolysis and promote M1 macrophage activation via the PPAR-γ/NF-κB pathway. Both silencing CTRP6 expression and treatment with the PPAR-γ agonist GW1929 can reverse the M1 phenotype effects *in vitro* and *in vivo* through decreasing glycolysis and blocking M1 macrophage polarization (122).

In addition to associated molecules, some drugs also help improve obesity and obesity-related metabolic disorders. Sulforaphane is present in a variety of cruciferous vegetables and has numerous functions. Sulforaphane exhibits a distinct transcriptional pathway that protects against obesity by lowering fatty acid synthesis, promoting ribosome biogenesis and reducing ROS accumulation (123). A recent study demonstrated that sulforaphane plays a role in activating M2 macrophage polarization while inhibiting M1 macrophage polarization, which protects against abnormal extracellular matrix deposition (124). The popular thiazolidinedione pioglitazone has been demonstrated to be effective in preventing cardiovascular problems associated with type 2 diabetes. A study revealed that pioglitazone does not affect cell viability or macrophage differentiation but instead suppress CXCR7 expression, blocking chemotaxis in differentiated macrophages. Furthermore, pioglitazone-induced CXCR7 suppression and chemotaxis inhibition occur through the activation of peroxisome PPARγ in differentiated macrophages (125).

There are also many anti-inflammatory medicines that can reduce cytokine secretion. The primary signaling mechanisms for TNF-α-mediated inflammation involve the NF-κB and MAPK pathways. Cimifugin is a common component of traditional Chinese herbs that can combat inflammatory diseases. It protects against oxidative stress and inflammation by inhibiting the NF-κB/MAPK signaling pathway (126). In 3T3-L1 adipocytes, cimifugin decreases the synthesis of proinflammatory factors and phospho-P65 expression, as well as the activation of the MAPK pathway and accumulation of intracellular lipids (127). In addition, TNF-α-blocking peptides can play a similar role. SN1-13 inhibits TNF-α-mediated signaling by preventing TNF-α and its receptors, and it can regulate the production of inflammatory mediators, including TNF-α, IL-1β, IL-6, and IFN-γ (128). In addition, atorvastatin can reduce CXCR7 mRNA and protein expression and prevent CXCR7 activation to inhibit macrophage migration (129). Although these medicines are not reported for use in the treatment of obesity, inhibiting the expression of proinflammatory cytokines seems to be beneficial for the treatment of obese patients. 

### 10.2 Targeting mitochondria in ATMs

Importantly, mitochondria play a role in thermogenesis, and their structure promotes or inhibits heat production. The mitochondrial calcium uniporter (Mcu) is a multimeric channel in the inner membrane that transports Ca^2+^ to the mitochondrial matrix. Inhibiting Mcu decreases adipocyte differentiation and lipid accumulation, alleviates high-fat diet induced metabolic abnormalities, and increases energy expenditure and thermogenesis (130). Similarly, overexpression of solute carrier family 25 member 28 (SLC25A28), an iron transporter located in the inner mitochondrial membrane, can reduce the development of BAT by downregulating PGC-1α and UCP1, leading to lipolysis inhibition and lipid accumulation (131). Phosphoglycerate mutase member 5 (PGAM5) locating at the mitochondrial outer membrane can be a potential therapeutic target because it can modulate the activation of downstream signaling pathways, such as the NF-κB and MAPK pathways, which in turn controls the expression and synthesis of inflammatory cytokines in macrophages (132).

Activation of PPAR-α and dual PPARα/γ led to the recruitment of UCP1^+^ beige adipocytes and promoted UCP1-independent thermogenesis, resulting in normalized body mass and insulin sensitivity levels (133). A study revealed that miR-155 produced by obese ATMs influences the insulin signaling pathway by targeting PPAR-γ (134). In addition, a recent study revealed that ATM-derived miR-210-3p-enriched EVs increase glucose intolerance, intensify systemic IR, and decrease glucose uptake in adipocytes by directly suppressing GLUT4 expression. However, targeted suppression of miR-210-3p prevents glucose intolerance and insulin resistance in HFD-fed mice (135).

## 11 Conclusions and perspective

Obesity is a global disease that causes many metabolic diseases. The state of inflammation and decreased energy expenditure are the two most significant features of obesity. Increasing evidence has demonstrated that macrophages participate largely in the development of obesity and inflammation. In obese mice, M2 macrophages are diverted into the M1 macrophage state, which secretes many inflammatory cytokines, causing a systemic and chronic inflammatory state. However, in addition to their inflammatory role, macrophages also perform a phagocytosis function and regulate lipogenesis. Fat accumulation and macrophage changes mutually influence each other. Moreover, mitochondrial dysfunction occurs in both macrophages and adipocytes. Overall, not only macrophage polarization or recruitment but also mitochondria can be potential therapeutic targets for obesity, but further research is needed.

## References

[1] Whitlock G Lewington S Sherliker P Clarke R Emberson J Halsey J. Body-mass index and cause-specific mortality in 900 000 adults: collaborative analyses of 57 prospective studies. Lancet (London England). (2009) 373:1083–96. doi: 10.1016/S0140-6736(09)60318-4 PMC2662372 19299006

[2] Nowell HP Rosie KS Bin Z Rachel AH Anu M James EB. Worldwide trends in underweight and obesity from 1990 to 2022: a pooled analysis of 3663 population-representative studies with 222 million children, adolescents, and adults. Lancet (London England). (2024) 403:1027–50. doi: 10.1016/S0140-6736(23)02750-2 PMC7615769 38432237

[3] Gobato AO Vasques AC Zambon MP Barros Filho Ade A Hessel G. Metabolic syndrome and insulin resistance in obese adolescents. Rev paulista pediatria: orgao oficial da Sociedade Pediatria Sao Paulo. (2014) 32:55–62. doi: 10.1590/S0103-05822014000100010 PMC4182990 24676191

[4] Litwin M Kułaga Z. Obesity, metabolic syndrome, and primary hypertension. Pediatr Nephrol (Berlin Germany). (2021) 36:825–37. doi: 10.1007/s00467-020-04579-3 PMC7910261 32388582

[5] Berrington de Gonzalez A Hartge P Cerhan JR Flint AJ Hannan L MacInnis RJ. Body-mass index and mortality among 1. 46 million white adults. New Engl J Med. (2010) 363:2211–9. doi: 10.1056/NEJMoa1000367 PMC3066051 21121834

[6] Calle EE. Obesity and cancer. BMJ (Clinical Res ed). (2007) 335:1107–8. doi: 10.1136/bmj.39384.472072.80 PMC2099565 17986715

[7] Dong SS Zhang K Guo Y Ding JM Rong Y Feng JC. Phenome-wide investigation of the causal associations between childhood BMI and adult trait outcomes: a two-sample Mendelian randomization study. Genome Med. (2021) 13:48. doi: 10.1186/s13073-021-00865-3 33771188 PMC8004431

[8] Blüher M. Obesity: global epidemiology and pathogenesis. Nat Rev Endocrinology. (2019) 15:288–98. doi: 10.1038/s41574-019-0176-8 30814686

[9] Bray GA Ryan DH. Evidence-based weight loss interventions: Individualized treatment options to maximize patient outcomes. Diabetes Obes Metab. (2021) 23 Suppl 1:50–62. doi: 10.1111/dom.14200 32969147

[10] Sartipy P Loskutoff DJ. Monocyte chemoattractant protein 1 in obesity and insulin resistance. Proc Natl Acad Sci United States America. (2003) 100:7265–70. doi: 10.1073/pnas.1133870100 PMC165864 12756299

[11] O'Sullivan J Lysaght J Donohoe CL Reynolds JV. Obesity and gastrointestinal cancer: the interrelationship of adipose and tumour microenvironments. Nat Rev Gastroenterol hepatology. (2018) 15:699–714. doi: 10.1038/s41575-018-0069-7 30323319

[12] Cildir G Akıncılar SC Tergaonkar V. Chronic adipose tissue inflammation: all immune cells on the stage. Trends Mol Med. (2013) 19:487–500. doi: 10.1016/j.molmed.2013.05.001 23746697

[13] Heinonen S Jokinen R Rissanen A Pietiläinen KH. White adipose tissue mitochondrial metabolism in health and in obesity. Obes reviews: an Off J Int Assoc Study Obes. (2020) 21:e12958. doi: 10.1111/obr.12958 31777187

[14] Müller TD Blüher M Tschöp MH DiMarchi RD. Anti-obesity drug discovery: advances and challenges. Nat Rev Drug discovery. (2022) 21:201–23. doi: 10.1038/s41573-021-00337-8 PMC8609996 34815532

[15] Ferrante AW Jr. Macrophages, fat, and the emergence of immunometabolism. J Clin Invest. (2013) 123:4992–3. doi: 10.1172/JCI73658 PMC3859415 24292661

[16] Chavakis T Alexaki VI Ferrante AW Jr. Macrophage function in adipose tissue homeostasis and metabolic inflammation. Nat Immunol. (2023) 24:757–66. doi: 10.1038/s41590-023-01479-0 37012544

[17] Jiang Y Liang M Chen L Wang J Huang Y Huo H. Myeloid SENP3 deficiency protects mice from diet and age-induced obesity via regulation of YAP1 SUMOylation. Cell Mol Life sciences: CMLS. (2023) 81:4. doi: 10.1007/s00018-023-05050-w 38070059 PMC10710392

[18] Yanovski JA. Obesity: Trends in underweight and obesity - scale of the problem. Nat Rev Endocrinology. (2018) 14:5–6. doi: 10.1038/nrendo.2017.157 29170540 PMC5800307

[19] Scuteri A Sanna S Chen WM Uda M Albai G Strait J. Genome-wide association scan shows genetic variants in the FTO gene are associated with obesity-related traits. PloS Genet. (2007) 3:e115. doi: 10.1371/journal.pgen.0030115 17658951 PMC1934391

[20] Akbari P Gilani A Sosina O Kosmicki JA Khrimian L Fang YY. Sequencing of 640,000 exomes identifies GPR75 variants associated with protection from obesity. Sci (New York NY). (2021) 373:eabf8683. doi: 10.1126/science.abf8683 PMC10275396 34210852

[21] Kaisinger LR Kentistou KA Stankovic S Gardner EJ Day FR Zhao Y. Large-scale exome sequence analysis identifies sex- and age-specific determinants of obesity. Cell Genomics. (2023) 3:100362. doi: 10.1016/j.xgen.2023.100362 37601970 PMC10435378

[22] Zhang Y Proenca R Maffei M Barone M Leopold L Friedman JM. Positional cloning of the mouse obese gene and its human homologue. Nature. (1994) 372:425–32. doi: 10.1038/372425a0 7984236

[23] Krude H Biebermann H Luck W Horn R Brabant G Grüters A. Severe early-onset obesity, adrenal insufficiency and red hair pigmentation caused by POMC mutations in humans. Nat Genet. (1998) 19:155–7. doi: 10.1038/509 9620771

[24] Clément K Vaisse C Lahlou N Cabrol S Pelloux V Cassuto D. A mutation in the human leptin receptor gene causes obesity and pituitary dysfunction. Nature. (1998) 392:398–401. doi: 10.1038/32911 9537324

[25] Farooqi IS Yeo GS Keogh JM Aminian S Jebb SA Butler G. Dominant and recessive inheritance of morbid obesity associated with melanocortin 4 receptor deficiency. J Clin Invest. (2000) 106:271–9. doi: 10.1172/JCI9397 PMC314308 10903343

[26] Lee SJ Shin SW. Mechanisms, pathophysiology, and management of obesity. New Engl J Med. (2017) 376:1491–2. doi: 10.1056/NEJMc1701944 28406283

[27] McHill AW Wright KP Jr. Role of sleep and circadian disruption on energy expenditure and in metabolic predisposition to human obesity and metabolic disease. Obes reviews: an Off J Int Assoc Study Obes. (2017) 18 Suppl 1:15–24. doi: 10.1111/obr.12503 28164449

[28] Wilkinson MJ Manoogian ENC Zadourian A Lo H Fakhouri S Shoghi A. Ten-hour time-restricted eating reduces weight, blood pressure, and atherogenic lipids in patients with metabolic syndrome. Cell Metab. (2020) 31:92–104.e5. doi: 10.1016/j.cmet.2019.11.004 31813824 PMC6953486

[29] Kershaw EE Flier JS. Adipose tissue as an endocrine organ. J Clin Endocrinol Metab. (2004) 89:2548–56. doi: 10.1210/jc.2004-0395 15181022

[30] Talukdar S Oh DY Bandyopadhyay G Li D Xu J McNelis J. Neutrophils mediate insulin resistance in mice fed a high-fat diet through secreted elastase. Nat Med. (2012) 18:1407–12. doi: 10.1038/nm.2885 PMC3491143 22863787

[31] Chatzigeorgiou A Karalis KP Bornstein SR Chavakis T. Lymphocytes in obesity-related adipose tissue inflammation. Diabetologia. (2012) 55:2583–92. doi: 10.1007/s00125-012-2607-0 22733483

[32] Wu D Molofsky AB Liang HE Ricardo-Gonzalez RR Jouihan HA Bando JK. Eosinophils sustain adipose alternatively activated macrophages associated with glucose homeostasis. Sci (New York NY). (2011) 332:243–7. doi: 10.1126/science.1201475 PMC3144160 21436399

[33] Brestoff JR Kim BS Saenz SA Stine RR Monticelli LA Sonnenberg GF. Group 2 innate lymphoid cells promote beiging of white adipose tissue and limit obesity. Nature. (2015) 519:242–6. doi: 10.1038/nature14115 PMC4447235 25533952

[34] Lumeng CN Bodzin JL Saltiel AR. Obesity induces a phenotypic switch in adipose tissue macrophage polarization. J Clin Invest. (2007) 117:175–84. doi: 10.1172/JCI29881 PMC1716210 17200717

[35] Lumeng CN DelProposto JB Westcott DJ Saltiel AR. Phenotypic switching of adipose tissue macrophages with obesity is generated by spatiotemporal differences in macrophage subtypes. Diabetes. (2008) 57:3239–46. doi: 10.2337/db08-0872 PMC2584129 18829989

[36] Biswas SK Mantovani A. Macrophage plasticity and interaction with lymphocyte subsets: cancer as a paradigm. Nat Immunol. (2010) 11:889–96. doi: 10.1038/ni.1937 20856220

[37] Chawla A Nguyen KD Goh YP. Macrophage-mediated inflammation in metabolic disease. Nat Rev Immunol. (2011) 11:738–49. doi: 10.1038/nri3071 PMC3383854 21984069

[38] Atri C Guerfali FZ Laouini D. Role of human macrophage polarization in inflammation during infectious diseases. Int J Mol Sci. (2018) 19:1801. doi: 10.3390/ijms19061801 29921749 PMC6032107

[39] Jiang Z Zhu L. Update on the role of alternatively activated macrophages in asthma. J Asthma Allergy. (2016) 9:101–7. doi: 10.2147/JAA.S104508 PMC4902247 27350756

[40] Colin S Chinetti-Gbaguidi G Staels B. Macrophage phenotypes in atherosclerosis. Immunol Rev. (2014) 262:153–66. doi: 10.1111/imr.2014.262.issue-1 25319333

[41] Zeyda M Gollinger K Kriehuber E Kiefer FW Neuhofer A Stulnig TM. Newly identified adipose tissue macrophage populations in obesity with distinct chemokine and chemokine receptor expression. Int J Obes (2005). (2010) 34:1684–94. doi: 10.1038/ijo.2010.103 20514049

[42] Morris DL Singer K Lumeng CN. Adipose tissue macrophages: phenotypic plasticity and diversity in lean and obese states. Curr Opin Clin Nutr Metab Care. (2011) 14:341–6. doi: 10.1097/MCO.0b013e328347970b PMC4690541 21587064

[43] Wentworth JM Naselli G Brown WA Doyle L Phipson B Smyth GK. Pro-inflammatory CD11c+CD206+ adipose tissue macrophages are associated with insulin resistance in human obesity. Diabetes. (2010) 59:1648–56. doi: 10.2337/db09-0287 PMC2889764 20357360

[44] Chapman K Holmes M Seckl J. 11β-hydroxysteroid dehydrogenases: intracellular gate-keepers of tissue glucocorticoid action. Physiol Rev. (2013) 93:1139–206. doi: 10.1152/physrev.00020.2012 PMC3962546 23899562

[45] Nakajima S Koh V Kua LF So J Davide L Lim KS. Accumulation of CD11c+CD163+ Adipose tissue macrophages through upregulation of intracellular 11β-HSD1 in human obesity. J Immunol (Baltimore Md: 1950). (2016) 197:3735–45. doi: 10.4049/jimmunol.1600895 27698011

[46] Jaitin DA Adlung L Thaiss CA Weiner A Li B Descamps H. Lipid-associated macrophages control metabolic homeostasis in a trem2-dependent manner. Cell. (2019) 178:686–98.e14. doi: 10.1016/j.cell.2019.05.054 31257031 PMC7068689

[47] Hill DA Lim HW Kim YH Ho WY Foong YH Nelson VL. Distinct macrophage populations direct inflammatory versus physiological changes in adipose tissue. Proc Natl Acad Sci United States America. (2018) 115:E5096–e105. doi: 10.1073/pnas.1802611115 PMC5984532 29760084

[48] Kratz M Coats BR Hisert KB Hagman D Mutskov V Peris E. Metabolic dysfunction drives a mechanistically distinct proinflammatory phenotype in adipose tissue macrophages. Cell Metab. (2014) 20:614–25. doi: 10.1016/j.cmet.2014.08.010 PMC4192131 25242226

[49] Xu X Grijalva A Skowronski A van Eijk M Serlie MJ Ferrante AW Jr. Obesity activates a program of lysosomal-dependent lipid metabolism in adipose tissue macrophages independently of classic activation. Cell Metab. (2013) 18:816–30. doi: 10.1016/j.cmet.2013.11.001 PMC3939841 24315368

[50] Haka AS Barbosa-Lorenzi VC Lee HJ Falcone DJ Hudis CA Dannenberg AJ. Exocytosis of macrophage lysosomes leads to digestion of apoptotic adipocytes and foam cell formation. J Lipid Res. (2016) 57:980–92. doi: 10.1194/jlr.M064089 PMC4878183 27044658

[51] Oh H Park SH Kang MK Kim YH Lee EJ Kim DY. Asaronic Acid Attenuates Macrophage Activation toward M1 Phenotype through Inhibition of NF-κB Pathway and JAK-STAT Signaling in Glucose-Loaded Murine Macrophages. J Agric Food Chem. (2019) 67:10069–78. doi: 10.1021/acs.jafc.9b03926 31422663

[52] Sawoo R Bishayi B. TLR4/TNFR1 blockade suppresses STAT1/STAT3 expression and increases SOCS3 expression in modulation of LPS-induced macrophage responses. Immunobiology. (2024) 229:152840. doi: 10.1016/j.imbio.2024.152840 39126792

[53] Hu X Herrero C Li WP Antoniv TT Falck-Pedersen E Koch AE. Sensitization of IFN-gamma Jak-STAT signaling during macrophage activation. Nat Immunol. (2002) 3:859–66. doi: 10.1038/ni828 12172544

[54] Krausgruber T Blazek K Smallie T Alzabin S Lockstone H Sahgal N. IRF5 promotes inflammatory macrophage polarization and TH1-TH17 responses. Nat Immunol. (2011) 12:231–8. doi: 10.1038/ni.1990 21240265

[55] Chrysafi P Perakakis N Farr OM Stefanakis K Peradze N Sala-Vila A. Leptin alters energy intake and fat mass but not energy expenditure in lean subjects. Nat Commun. (2020) 11:5145. doi: 10.1038/s41467-020-18885-9 33051459 PMC7553922

[56] Wang Y Wan R Hu C. Leptin/obR signaling exacerbates obesity-related neutrophilic airway inflammation through inflammatory M1 macrophages. Mol Med (Cambridge Mass). (2023) 29:100. doi: 10.1186/s10020-023-00702-w 37488474 PMC10367413

[57] Nishimura S Manabe I Nagasaki M Eto K Yamashita H Ohsugi M. CD8+ effector T cells contribute to macrophage recruitment and adipose tissue inflammation in obesity. Nat Med. (2009) 15:914–20. doi: 10.1038/nm.1964 19633658

[58] Lord GM Matarese G Howard JK Baker RJ Bloom SR Lechler RI. Leptin modulates the T-cell immune response and reverses starvation-induced immunosuppression. Nature. (1998) 394:897–901. doi: 10.1038/29795 9732873

[59] O'Rourke RW Meyer KA Neeley CK Gaston GD Sekhri P Szumowski M. Systemic NK cell ablation attenuates intra-abdominal adipose tissue macrophage infiltration in murine obesity. Obes (Silver Spring Md). (2014) 22:2109–14. doi: 10.1002/oby.20823 PMC4180782 24962029

[60] Williams L Bradley L Smith A Foxwell B. Signal transducer and activator of transcription 3 is the dominant mediator of the anti-inflammatory effects of IL-10 in human macrophages. J Immunol (Baltimore Md: 1950). (2004) 172:567–76. doi: 10.4049/jimmunol.172.1.567 14688368

[61] Finbloom DS Winestock KD. IL-10 induces the tyrosine phosphorylation of tyk2 and Jak1 and the differential assembly of STAT1 alpha and STAT3 complexes in human T cells and monocytes. J Immunol (Baltimore Md: 1950). (1995) 155:1079–90. doi: 10.4049/jimmunol.155.3.1079 7543512

[62] Sica A Mantovani A. Macrophage plasticity and polarization: in *vivo* veritas. J Clin Invest. (2012) 122:787–95. doi: 10.1172/JCI59643 PMC3287223 22378047

[63] Feng L Li C Zeng LW Gao D Sun YH Zhong L. MARCH3 negatively regulates IL-3-triggered inflammatory response by mediating K48-linked polyubiquitination and degradation of IL-3Rα. Signal transduction targeted Ther. (2022) 7:21. doi: 10.1038/s41392-021-00834-7 PMC8786845 35075102

[64] Martinez FO Helming L Gordon S. Alternative activation of macrophages: an immunologic functional perspective. Annu Rev Immunol. (2009) 27:451–83. doi: 10.1146/annurev.immunol.021908.132532 19105661

[65] Turer AT Scherer PE. Adiponectin: mechanistic insights and clinical implications. Diabetologia. (2012) 55:2319–26. doi: 10.1007/s00125-012-2598-x 22688349

[66] Xuan D Han Q Tu Q Zhang L Yu L Murry D. Epigenetic modulation in periodontitis: interaction of adiponectin and JMJD3-IRF4 axis in macrophages. J Cell Physiol. (2016) 231:1090–6. doi: 10.1002/jcp.v231.5 PMC5298882 26399931

[67] Tilg H Moschen AR. Adipocytokines: mediators linking adipose tissue, inflammation and immunity. Nat Rev Immunol. (2006) 6:772–83. doi: 10.1038/nri1937 16998510

[68] Li C Ying W Huang Z Brehm T Morin A Vella AT. IRF6 regulates alternative activation by suppressing PPARγ in male murine macrophages. Endocrinology. (2017) 158:2837–47. doi: 10.1210/en.2017-00053 PMC5659664 28645193

[69] Lin Y Zhao JL Zheng QJ Jiang X Tian J Liang SQ. Notch signaling modulates macrophage polarization and phagocytosis through direct suppression of signal regulatory protein α Expression. Front Immunol. (2018) 9:1744. doi: 10.3389/fimmu.2018.01744 30105024 PMC6077186

[70] Tang D Han B He C Xu Y Liu Z Wang W. Electrospun poly-l-lactic acid membranes promote M2 macrophage polarization by regulating the PCK2/AMPK/mTOR signaling pathway. Advanced Healthcare Materials. (2024) 13: e2400481. doi: 10.1002/adhm.202400481 38650356

[71] Mounier R Théret M Arnold L Cuvellier S Bultot L Göransson O. AMPKα1 regulates macrophage skewing at the time of resolution of inflammation during skeletal muscle regeneration. Cell Metab. (2013) 18:251–64. doi: 10.1016/j.cmet.2013.06.017 23931756

[72] Galic S Fullerton MD Schertzer JD Sikkema S Marcinko K Walkley CR. Hematopoietic AMPK β1 reduces mouse adipose tissue macrophage inflammation and insulin resistance in obesity. J Clin Invest. (2011) 121:4903–15. doi: 10.1172/JCI58577 PMC3226000 22080866

[73] Weisberg SP McCann D Desai M Rosenbaum M Leibel RL Ferrante AW Jr. Obesity is associated with macrophage accumulation in adipose tissue. J Clin Invest. (2003) 112:1796–808. doi: 10.1172/JCI200319246 PMC296995 14679176

[74] Murano I Barbatelli G Parisani V Latini C Muzzonigro G Castellucci M. Dead adipocytes, detected as crown-like structures, are prevalent in visceral fat depots of genetically obese mice. J Lipid Res. (2008) 49:1562–8. doi: 10.1194/jlr.M800019-JLR200 18390487

[75] Suganami T Nishida J Ogawa Y. A paracrine loop between adipocytes and macrophages aggravates inflammatory changes: role of free fatty acids and tumor necrosis factor alpha. Arteriosclerosis thrombosis Vasc Biol. (2005) 25:2062–8. doi: 10.1161/01.ATV.0000183883.72263.13 16123319

[76] Matsushima K Larsen CG DuBois GC Oppenheim JJ. Purification and characterization of a novel monocyte chemotactic and activating factor produced by a human myelomonocytic cell line. J Exp Med. (1989) 169:1485–90. doi: 10.1084/jem.169.4.1485 PMC2189236 2926331

[77] Kanda H Tateya S Tamori Y Kotani K Hiasa K Kitazawa R. MCP-1 contributes to macrophage infiltration into adipose tissue, insulin resistance, and hepatic steatosis in obesity. J Clin Invest. (2006) 116:1494–505. doi: 10.1172/JCI26498 PMC1459069 16691291

[78] Hu JK Kagari T Clingan JM Matloubian M. Expression of chemokine receptor CXCR3 on T cells affects the balance between effector and memory CD8 T-cell generation. Proc Natl Acad Sci United States America. (2011) 108:E118–27. doi: 10.1073/pnas.1101881108 PMC3102421 21518913

[79] Kiran S Kumar V Murphy EA Enos RT Singh UP. High fat diet-induced CD8(+) T cells in adipose tissue mediate macrophages to sustain low-grade chronic inflammation. Front Immunol. (2021) 12:680944. doi: 10.3389/fimmu.2021.680944 34248964 PMC8261297

[80] Deiuliis JA Oghumu S Duggineni D Zhong J Rutsky J Banerjee A. CXCR3 modulates obesity-induced visceral adipose inflammation and systemic insulin resistance. Obes (Silver Spring Md). (2014) 22:1264–74. doi: 10.1002/oby.20642 PMC4167757 24124129

[81] Tourniaire F Romier-Crouzet B Lee JH Marcotorchino J Gouranton E Salles J. Chemokine expression in inflamed adipose tissue is mainly mediated by NF-κB. PloS One. (2013) 8:e66515. doi: 10.1371/journal.pone.0066515 23824685 PMC3688928

[82] Ma W Liu Y Ellison N Shen J. Induction of C-X-C chemokine receptor type 7 (CXCR7) switches stromal cell-derived factor-1 (SDF-1) signaling and phagocytic activity in macrophages linked to atherosclerosis. J Biol Chem. (2013) 288:15481–94. doi: 10.1074/jbc.M112.445510 PMC3668710 23599431

[83] Peng H Zhang H Zhu H. Blocking CXCR7-mediated adipose tissue macrophages chemotaxis attenuates insulin resistance and inflammation in obesity. Biochem Biophys Res Commun. (2016) 479:649–55. doi: 10.1016/j.bbrc.2016.09.158 27693695

[84] Cannon B Nedergaard J. Brown adipose tissue: function and physiological significance. Physiol Rev. (2004) 84:277–359. doi: 10.1152/physrev.00015.2003 14715917

[85] Wu J Boström P Sparks LM Ye L Choi JH Giang AH. Beige adipocytes are a distinct type of thermogenic fat cell in mouse and human. Cell. (2012) 150:366–76. doi: 10.1016/j.cell.2012.05.016 PMC3402601 22796012

[86] Gaspar RS Delafiori J Zuccoli G Carregari VC Prado TP Morari J. Exogenous succinate impacts mouse brown adipose tissue mitochondrial proteome and potentiates body mass reduction induced by liraglutide. Am J Physiol Endocrinol Metab. (2023) 324:E226–e40. doi: 10.1152/ajpendo.00231.2022 36724126

[87] Zhang W Wang S Liu Z Qian P Li Y Wu J. Legumain-deficient macrophages regulate inflammation and lipid metabolism in adipose tissues to protect against diet-induced obesity. Mol Cell endocrinology. (2024) 592:112283. doi: 10.1016/j.mce.2024.112283 38815795

[88] Lund C Ranea-Robles P Falk S Rausch DM Skovbjerg G Vibe-Petersen VK. Protection against overfeeding-induced weight gain is preserved in obesity but does not require FGF21 or MC4R. Nat Commun. (2024) 15:1192. doi: 10.1038/s41467-024-45223-0 38331907 PMC10853283

[89] Ryan DG Murphy MP Frezza C Prag HA Chouchani ET O'Neill LA. Coupling Krebs cycle metabolites to signalling in immunity and cancer. Nat Metab. (2019) 1:16–33. doi: 10.1038/s42255-018-0014-7 31032474 PMC6485344

[90] Vats D Mukundan L Odegaard JI Zhang L Smith KL Morel CR. Oxidative metabolism and PGC-1beta attenuate macrophage-mediated inflammation. Cell Metab. (2006) 4:13–24. doi: 10.1016/j.cmet.2006.05.011 16814729 PMC1904486

[91] Chi Z Chen S Xu T Zhen W Yu W Jiang D. Histone deacetylase 3 couples mitochondria to drive IL-1β-dependent inflammation by configuring fatty acid oxidation. Mol Cell. (2020) 80:43–58.e7. doi: 10.1016/j.molcel.2020.08.015 32937100

[92] Sonoda J Mehl IR Chong LW Nofsinger RR Evans RM. PGC-1beta controls mitochondrial metabolism to modulate circadian activity, adaptive thermogenesis, and hepatic steatosis. Proc Natl Acad Sci United States America. (2007) 104:5223–8. doi: 10.1073/pnas.0611623104 PMC1829290 17360356

[93] Chen H Liu Y Li D Song J Xia M. PGC-1β suppresses saturated fatty acid-induced macrophage inflammation by inhibiting TAK1 activation. IUBMB Life. (2016) 68:145–55. doi: 10.1002/iub.v68.2 26748475

[94] Toh ML Gonzales G Koenders MI Tournadre A Boyle D Lubberts E. Role of interleukin 17 in arthritis chronicity through survival of synoviocytes via regulation of synoviolin expression. PloS One. (2010) 5:e13416. doi: 10.1371/journal.pone.0013416 20976214 PMC2955522

[95] Fujita H Yagishita N Aratani S Saito-Fujita T Morota S Yamano Y. The E3 ligase synoviolin controls body weight and mitochondrial biogenesis through negative regulation of PGC-1β. EMBO J. (2015) 34:1042–55. doi: 10.15252/embj.201489897 PMC4406651 25698262

[96] Acín-Pérez R Iborra S Martí-Mateos Y Cook ECL Conde-Garrosa R Petcherski A. Fgr kinase is required for proinflammatory macrophage activation during diet-induced obesity. Nat Metab. (2020) 2:974–88. doi: 10.1038/s42255-020-00273-8 PMC8225238 32943786

[97] Pridans C Raper A Davis GM Alves J Sauter KA Lefevre L. Pleiotropic impacts of macrophage and microglial deficiency on development in rats with targeted mutation of the csf1r locus. J Immunol (Baltimore Md: 1950). (2018) 201:2683–99. doi: 10.4049/jimmunol.1701783 PMC6196293 30249809

[98] Cox N Crozet L Holtman IR Loyher PL Lazarov T White JB. Diet-regulated production of PDGFcc by macrophages controls energy storage. Sci (New York NY). (2021) 373:eabe9383. doi: 10.1126/science.abe9383 PMC9558257 34210853

[99] Pirzgalska RM Seixas E Seidman JS Link VM Sánchez NM Mahú I. Sympathetic neuron-associated macrophages contribute to obesity by importing and metabolizing norepinephrine. Nat Med. (2017) 23:1309–18. doi: 10.1038/nm.4422 PMC7104364 29035364

[100] Tormos KV Anso E Hamanaka RB Eisenbart J Joseph J Kalyanaraman B. Mitochondrial complex III ROS regulate adipocyte differentiation. Cell Metab. (2011) 14:537–44. doi: 10.1016/j.cmet.2011.08.007 PMC3190168 21982713

[101] Liesa M Shirihai OS. Mitochondrial dynamics in the regulation of nutrient utilization and energy expenditure. Cell Metab. (2013) 17:491–506. doi: 10.1016/j.cmet.2013.03.002 23562075 PMC5967396

[102] Hernández-Aguilera A Rull A Rodríguez-Gallego E Riera-Borrull M Luciano-Mateo F Camps J. Mitochondrial dysfunction: a basic mechanism in inflammation-related non-communicable diseases and therapeutic opportunities. Mediators inflammation. (2013) 2013:135698. doi: 10.1155/2013/135698 PMC3603328 23533299

[103] Yin X Lanza IR Swain JM Sarr MG Nair KS Jensen MD. Adipocyte mitochondrial function is reduced in human obesity independent of fat cell size. J Clin Endocrinol Metab. (2014) 99:E209–16. doi: 10.1210/jc.2013-3042 PMC3913808 24276464

[104] Xia W Veeragandham P Cao Y Xu Y Rhyne TE Qian J. Obesity causes mitochondrial fragmentation and dysfunction in white adipocytes due to RalA activation. Nat Metab. (2024) 6:273–89. doi: 10.1038/s42255-024-00978-0 PMC10896723 38286821

[105] Hachiya K Deguchi Y Hirata T Arikawa T Fukai H Esashi T. (ZNF746) accumulation in adipose progenitor cells leads to attenuated mitochondrial biogenesis and impaired adipogenesis. Sci Rep. (2023) 13:22990. doi: 10.1038/s41598-023-49996-0 38151567 PMC10752882

[106] Scozzi D Ibrahim M Liao F Lin X Hsiao HM Hachem R. Mitochondrial damage-associated molecular patterns released by lung transplants are associated with primary graft dysfunction. Am J transplantation: Off J Am Soc Transplant Am Soc Transplant Surgeons. (2019) 19:1464–77. doi: 10.1111/ajt.15232 PMC6482093 30582269

[107] Hayakawa K Esposito E Wang X Terasaki Y Liu Y Xing C. Transfer of mitochondria from astrocytes to neurons after stroke. Nature. (2016) 535:551–5. doi: 10.1038/nature18928 PMC4968589 27466127

[108] Chang JC Chang HS Wu YC Cheng WL Lin TT Chang HJ. Mitochondrial transplantation regulates antitumour activity, chemoresistance and mitochondrial dynamics in breast cancer. J Exp Clin Cancer research: CR. (2019) 38:30. doi: 10.1186/s13046-019-1028-z 30674338 PMC6343292

[109] Dong LF Kovarova J Bajzikova M Bezawork-Geleta A Svec D Endaya B. Horizontal transfer of whole mitochondria restores tumorigenic potential in mitochondrial DNA-deficient cancer cells. eLife. (2017) 6:e22187. doi: 10.7554/eLife.22187 28195532 PMC5367896

[110] Brestoff JR Wilen CB Moley JR Li Y Zou W Malvin NP. Intercellular mitochondria transfer to macrophages regulates white adipose tissue homeostasis and is impaired in obesity. Cell Metab. (2021) 33:270–82.e8. doi: 10.1016/j.cmet.2020.11.008 33278339 PMC7858234

[111] Borcherding N Jia W Giwa R Field RL Moley JR Kopecky BJ. Dietary lipids inhibit mitochondria transfer to macrophages to divert adipocyte-derived mitochondria into the blood. Cell Metab. (2022) 34:1499–513.e8. doi: 10.1016/j.cmet.2022.08.010 36070756 PMC9547954

[112] Rosina M Ceci V Turchi R Chuan L Borcherding N Sciarretta F. Ejection of damaged mitochondria and their removal by macrophages ensure efficient thermogenesis in brown adipose tissue. Cell Metab. (2022) 34:533–48.e12. doi: 10.1016/j.cmet.2022.02.016 35305295 PMC9039922

[113] Wang YN Tang Y He Z Ma H Wang L Liu Y. Slit3 secreted from M2-like macrophages increases sympathetic activity and thermogenesis in adipose tissue. Nat Metab. (2021) 3:1536–51. doi: 10.1038/s42255-021-00482-9 34782792

[114] Sakamoto T Takahashi N Sawaragi Y Naknukool S Yu R Goto T. Inflammation induced by RAW macrophages suppresses UCP1 mRNA induction via ERK activation in 10T1/2 adipocytes. Am J Physiol Cell Physiol. (2013) 304:C729–38. doi: 10.1152/ajpcell.00312.2012 PMC3625802 23302779

[115] Goto T Naknukool S Yoshitake R Hanafusa Y Tokiwa S Li Y. Proinflammatory cytokine interleukin-1β suppresses cold-induced thermogenesis in adipocytes. Cytokine. (2016) 77:107–14. doi: 10.1016/j.cyto.2015.11.001 26556104

[116] Rajbhandari P Thomas BJ Feng AC Hong C Wang J Vergnes L. IL-10 signaling remodels adipose chromatin architecture to limit thermogenesis and energy expenditure. Cell. (2018) 172:218–33.e17. doi: 10.1016/j.cell.2017.11.019 29249357 PMC5766418

[117] Wang Q Li D Cao G Shi Q Zhu J Zhang M. IL-27 signalling promotes adipocyte thermogenesis and energy expenditure. Nature. (2021) 600:314–8. doi: 10.1038/s41586-021-04127-5 34819664

[118] Chen SM Hsiao CW Chen YJ Hong CJ Lin JC Yang CP. Interleukin-4 inhibits the hypothalamic appetite control by modulating the insulin-AKT and JAK-STAT signaling in leptin mutant mice. Environ Toxicol. (2024) 39:3980–90. doi: 10.1002/tox.24264 38597583

[119] Wei D Tian X Ren Z Liu Z Sun C. Mechanistic insights into the role of USP14 in adipose tissue macrophage recruitment and insulin resistance in obesity. Int J Biol macromolecules. (2024) 267:131645. doi: 10.1016/j.ijbiomac.2024.131645 38631582

[120] Chen M Zhu J Luo H Mu W Guo L. The journey towards physiology and pathology: Tracing the path of neuregulin 4. Genes diseases. (2024) 11:687–700. doi: 10.1016/j.gendis.2023.03.021 37692526 PMC10491916

[121] Zhou Q Wang Y Lu Z Wang B Li L You M. Mitochondrial dysfunction caused by SIRT3 inhibition drives proinflammatory macrophage polarization in obesity. Obes (Silver Spring Md). (2023) 31:1050–63. doi: 10.1002/oby.23707 36894333

[122] Yan S Ding J Wang Z Zhang F Li J Zhang Y. CTRP6 regulates M1 macrophage polarization via the PPAR-γ/NF-κB pathway and reprogramming glycolysis in recurrent spontaneous abortion. Int Immunopharmacol. (2023) 124:110840. doi: 10.1016/j.intimp.2023.110840 37696144

[123] Çakır I Lining Pan P Hadley CK El-Gamal A Fadel A Elsayegh D. Sulforaphane reduces obesity by reversing leptin resistance. eLife. (2022) 11. doi: 10.7554/eLife.67368 PMC8947770 35323110

[124] Zhang Z Chen H Pan C Li R Zhao W Song T. Sulforaphane reduces adipose tissue fibrosis via promoting M2 macrophages polarization in HFD fed-mice. Biochim Biophys Acta Mol Cell Res. (2024) 1871:119626. doi: 10.1016/j.bbamcr.2023.119626 37977492

[125] Zhao D Zhu Z Li D Xu R Wang T Liu K. Pioglitazone suppresses CXCR7 expression to inhibit human macrophage chemotaxis through peroxisome proliferator-activated receptor γ. Biochemistry. (2015) 54:6806–14. doi: 10.1021/acs.biochem.5b00847 26507929

[126] Liu A Zhao W Zhang B Tu Y Wang Q Li J. Cimifugin ameliorates imiquimod-induced psoriasis by inhibiting oxidative stress and inflammation via NF-κB/MAPK pathway. Bioscience Rep. (2020) 40:BSR20200471. doi: 10.1042/BSR20200471 PMC7300284 32515468

[127] Deng X Liu Z Han S. Cimifugin inhibits adipogenesis and TNF-α-induced insulin resistance in 3T3-L1 cells. Open Med (Warsaw Poland). (2023) 18:20230855. doi: 10.1515/med-2023-0855 PMC10693008 38045856

[128] Wang Y Ye R Fan L Zhao X Li L Zheng H. A TNF-α blocking peptide that reduces NF-κB and MAPK activity for attenuating inflammation. Bioorganic medicinal Chem. (2023) 92:117420. doi: 10.1016/j.bmc.2023.117420 37573821

[129] Ma W Liu Y Wang C Zhang L Crocker L Shen J. Atorvastatin inhibits CXCR7 induction to reduce macrophage migration. Biochem Pharmacol. (2014) 89:99–108. doi: 10.1016/j.bcp.2014.02.014 24582769

[130] Jia M Liu S Xiao Y Zhang Z Li M Qi X. Deletion of the mitochondrial calcium uniporter in adipose tissue promotes energy expenditure and alleviates diet-induced obesity. Mol Metab. (2024) 80:101873. doi: 10.1016/j.molmet.2024.101873 38199601 PMC10831290

[131] Guan H Xiao L Hao K Zhang Q Wu D Geng Z. SLC25A28 overexpression promotes adipogenesis by reducing ATGL. J Diabetes Res. (2024) 2024:5511454. doi: 10.1155/2024/5511454 38736904 PMC11088465

[132] Bang BR Miki H Kang YJ. Mitochondrial PGAM5-Drp1 signaling regulates the metabolic reprogramming of macrophages and regulates the induction of inflammatory responses. Front Immunol. (2023) 14:1243548. doi: 10.3389/fimmu.2023.1243548 37771598 PMC10523165

[133] Miranda CS Silva-Veiga FM Santana-Oliveira DA Vasques-Monteiro IML Daleprane JB Souza-Mello V. PPARα/γ synergism activates UCP1-dependent and -independent thermogenesis and improves mitochondrial dynamics in the beige adipocytes of high-fat fed mice. Nutr (Burbank Los Angeles County Calif). (2024) 117:112253. doi: 10.1016/j.nut.2023.112253 37944411

[134] Ying W Riopel M Bandyopadhyay G Dong Y Birmingham A Seo JB. Adipose tissue macrophage-derived exosomal miRNAs can modulate *in vivo* and *in vitro* insulin sensitivity. Cell. (2017) 171:372–84.e12. doi: 10.1016/j.cell.2017.08.035 28942920

[135] Patra D Ramprasad P Sharma S Dey U Kumar V Singh S. Adipose tissue macrophage-derived microRNA-210-3p disrupts systemic insulin sensitivity by silencing GLUT4 in obesity. J Biol Chem. (2024) 300:107328. doi: 10.1016/j.jbc.2024.107328 38679332 PMC11145551

